# Cost-effectiveness of prophylactic absorbable antibiotic cement for breast implant infection: A break-even analysis

**DOI:** 10.1016/j.jpra.2026.03.035

**Published:** 2026-04-03

**Authors:** Angad S. Sidhu, Shahnur Ahmed, Emma J. Bisch, Carla S. Fisher, Chris M. Reid, Aladdin H. Hassanein

**Affiliations:** a Division of Plastic Surgery, Indiana University School of Medicine, Indianapolis, IN United States of America; b Division of Breast Surgery, Indiana University School of Medicine, Indianapolis, IN United States of America; c Division of Plastic Surgery, University of California San Diego, San Diego, CA, United States of America

**Keywords:** Breast reconstruction, Tissue expander, Implant infection, Prophylactic absorbable antibiotic cement, Break-even, Cost-effective

## Abstract

Infection following prepectoral tissue expander (TE) reconstruction is challenging with reported rates between 10–15%. Traditional conservative management of significant TE infections includes intravenous antibiotics and explantation leading to delayed final breast reconstruction. Local delivery of antibiotics with absorbable calcium-sulfate cement following TE reconstruction recently has been shown to reduce TE infection rates. However, the cost-effectiveness of absorbable antibiotic cement routinely during breast reconstruction remains unclear. The purpose of this study is to evaluate and model infection rates, treatment costs, and pricing to determine the break-even threshold for prophylactic antibiotic cement placement. Absolute risk reduction (ARR) and number needed to break even (NNBE) were calculated across a range of infection rates and treatment costs. Using institutional cement cost of $1075 (USD), an estimated 10% infection rate, and an average $13,000 (USD) for an infection event, prophylactic cement becomes cost-effective when baseline infection rates exceed 8.3%, when infection management costs surpass $10,750, or when calcium-sulfate pricing remains below $1300. Non-selective prophylactic use may be economically justified under commonly reported clinical conditions and become even more favorable in settings with higher treatment costs or modestly lower cement pricing. A break-even analysis may be a valuable tool for evaluating infection-prevention strategies in TE-based reconstruction.

## Introduction

Postoperative infection remains a major challenge in prepectoral tissue expander (TE) breast reconstruction, leading to significant patient morbidity, reoperations, psychosocial distress, and increased healthcare costs.^1^ While techniques such as antibiotic irrigation and refined operative handling aim to reduce infection risk, interest is growing in local antimicrobial strategies. Absorbable calcium-sulfate antibiotic cement provides effective local drug delivery and has shown potential benefits in implant-based breast reconstruction.^1^ However, the cost-effectiveness of routine prophylactic antibiotic cement following prepectoral TE reconstruction remains unclear given variability in baseline infection rates and institutional cost structures. A break-even analysis provides a framework to determine the minimum absolute risk reduction required for cost neutrality based on bead pricing and infection management costs.^2^ This study applied a break-even model to define infection rate, treatment cost, and cement bead pricing thresholds under which prophylactic absorbable antibiotic cement becomes cost-effective in prepectoral TE reconstruction.

## Methods

A break-even analysis was performed using a validated economic equation to determine the cost-effective threshold for prophylactic absorbable antibiotic-impregnated calcium sulfate cement (5 mL, Stimulan, Biocomposites Ltd., UK) (Supplemental Figure 1).^2^ Absolute risk reduction (ARR) represented the reduction in infection rates required for cost neutrality. Baseline infection risk subtracted by ARR yielded the corresponding infection risk with antibiotic beads required for cost neutrality. The number needed to break even (NNBE) quantified how many patients could be treated before preventing one infection offset costs, with higher values indicating increased cost-effectiveness. This study used a combination of published literature and institutional cost data to estimate three key inputs: 1) baseline TE infection rates, 2) medical costs associated with treating a TE infection, and 3) antibiotic cement acquisition costs. These values informed calculations to determine break-even thresholds across a range of plausible clinical and cost scenarios.

## Results

The institutional cost of a 5-cc antibiotic cement kit was $1075. A baseline TE infection rate of 10% was used as a representative base-case estimate based on contemporary literature for prepectoral TE reconstruction (9–15%).^3^^,^^4^ Sensitivity analyses were performed across a range of infection risks to improve generalizability across institutions with differing complication rates (Table 1). The average cost of treating an infected TE was approximately $13,000.^5^ Prophylactic antibiotic cement beads broke even when the initial infection rate was above 8.3% (ARR: 8.3%, NNBE: 12). At 10% baseline infection risk, this ARR corresponds to an infection probability of approximately 1.7% with antibiotic beads. Therefore, up to 12 patients can be treated before preventing one infection offsets cost. When treatment costs exceeded $10,750, the cement became cost-effective at infection risks below 8.3% (Table 2). Sensitivity analyses demonstrated increased cost-effectiveness as the cost of treating an infection increased. Cement remained cost-effective below $1300 and at lower infection rates when treatment costs exceeded $10,750 (Supplemental Table 1).

**Table 1:** Cost-effectiveness of antibiotic cement at varying infection risk rates.

Initial infection rate	Final infection rate^b^	ARR^c^	NNBE^d^	Cost of Tx	Cost of beads
1%	—	8.30%	12	$13,000	$1075
5%	—	8.30%	12		
7%	—	8.30%	12		
8%	—	8.30%	12		
**8.30%**^a^	**0.00%**	8.30%	12		
9%	0.70%	8.30%	12		
10%	1.70%	8.30%	12		
12%	3.70%	8.30%	12		
15%	6.70%	8.30%	12		
20%	11.70%	8.30%	12		
25%	16.70%	8.30%	12		

a Break-even infection rate.

b Rows where baseline infection risk is lower than the ARR are shown as not clinically feasible (—) because infection risk cannot be negative.

c ARR (Absolute Risk Reduction): percentage by which the cement must reduce the initial infection rate by to break even.

d NNBE (Number Needed to Break Even): the higher the NNBE the more cost-effective the beads are as they need to prevent only 1 infection in a larger number of patients to be considered cost-effective.

**Table 2:** Cost-effectiveness of antibiotic cement with varying costs of treatment.

Initial infection rate	Cost of treatment	Final infection rate^b^	ARR	NNBE
10.00%	$1000	—	107.50%	1
	$2500	—	43.00%	2
	$5000	—	21.50%	5
	$7500	—	14.30%	7
	$10,000	—	10.80%	9
	**$10,750**^a^	**0.00%**	10.00%	10
	$11,000	0.23%	9.80%	10
	$15,000	2.83%	7.20%	14
	$20,000	4.63%	5.40%	19
	$30,000	6.42%	3.60%	28
	$50,000	7.85%	2.20%	47
	$75,000	8.57%	1.40%	70
	$100,000	8.93%	1.10%	93
	$250,000	9.57%	0.40%	233
	$500,000	9.79%	0.20%	465

a Break-even cost for treatment.

b Rows where baseline infection risk is lower than the ARR are shown as not clinically feasible (—) because infection risk cannot be negative.

## Discussion

Local delivery of prophylactic absorbable antibiotic cement has been shown to reduce TE infection, but economic value of routine use is unclear.^1^ This model suggests that prophylactic absorbable antibiotic cement breaks-even in TE-based reconstruction when 1) infection rates exceed 8.3%, 2) treatment costs exceed $10,750, or 3) when cement is priced under $1300. These thresholds typically fall within clinically observed ranges, indicating that modest reductions in infection risk may yield meaningful financial benefit given the high cost of managing postoperative complications. These findings demonstrate that prophylactic antibiotic cement may be financially justified under widely observed reconstructive conditions.

Break-even modeling is useful for evaluating infection-prevention economic value.^2^ Prior literature demonstrates the ARR required for cost-effectiveness is often smaller than the difference detectable in a clinical trial cohort, highlighting the relevance of economic modeling for interventions with expected, but difficult to quantify, benefits.^2^ Importantly, this model does not assume clinical effectiveness of antibiotic cement. Instead, it determines the minimum ARR required for cost neutrality. Observed clinical effectiveness can then be compared against this threshold to assess economic value. The ARR required for cost neutrality in our study is clinically relevant for TE-based reconstruction and may be achievable in most instances. The customization of inputs (i.e., rates, pricing, treatment cost) allows this model to provide a flexible framework to support decision-making around prophylactic antibiotic cement use across diverse settings. As healthcare systems shift toward value-based care, such models help evaluate interventions with expected but hard-to-measure benefits.

Importantly, institutional cost heterogeneity influences cost-effectiveness. Centers with higher operative or inpatient costs will reach the breakeven ARR at lower infection risk rates, supporting non-specific prophylactic usage. Additionally, published institutional costs likely underestimate the true downstream economic burden from payer or societal perspectives. Thus, the cost-effectiveness of prophylactic antibiotic cement may be even more favorable than demonstrated in this model. Our analysis supports the non-specific use of prophylactic antibiotic beads when identified thresholds are met. This model should be used by institutions to tailor prophylactic use of antibiotic beads during prepectoral TE reconstruction on their local event rates and resource structure.

## Declaration of competing interests

None declared.

## Appendix Supplementary materials

Image, application 1

Image, application 2
