# Supplementary material for: Cost-effectiveness of prophylactic absorbable antibiotic cement for breast implant infection: A break-even analysis
Source: JPRAS Open. 2026 Apr 3;49:509–12. doi: 10.1016/j.jpra.2026.03.035 (PMC13096895; doi:10.1016/j.jpra.2026.03.035)
Supplement: Supplementary file 1 [file mmc1.docx]

**SUPPLEMENTAL FIGURE 1**. Break-even equation to calculate cost-effectiveness of antibiotic cement beads; adapted from Hatch et al., 2017 ^[2]^

S_Total_ x C_Treatment_ x IR_Initial_ = (S_Total_ x C_Drug_) + (S_Total_ x C_Treatment_ x IR_Final_)

Solving for IR_Final_ yields:

IR_Final_ = ((IR_Final_ x C_Treatment_) - C_Drug_) / (C_Treatment_)

S_Total_ : Total annual procedures
C_Treatment_ : Cost of treatment
IR_Initial_ : Initial infection rate
C_Drug_ : Cost of drug
IR_Final_ : Final infection rate
