# Supplementary material for: Cost-effectiveness of prophylactic absorbable antibiotic cement for breast implant infection: A break-even analysis
Source: JPRAS Open. 2026 Apr 3;49:509–12. doi: 10.1016/j.jpra.2026.03.035 (PMC13096895; doi:10.1016/j.jpra.2026.03.035)
Supplement: Supplementary file 2 [file mmc2.docx]

***SUPPLEMENTAL MATERIAL***

**SUPPLEMENTAL TABLE 1.** Cost effectiveness based on varying prices of the antibiotic cement

| **Initial Infection Rate** | **Cost of Abx Bead** | **Final Infection Rate**** | **ARR** | **NNBE** |
| --- | --- | --- | --- | --- |
| 10.00% | $100.00 | 9.20% | 0.80% | 130 |
|  | $250.00 | 8.10% | 1.90% | 52 |
|  | $500.00 | 6.20% | 3.80% | 26 |
|  | $600.00 | 5.40% | 4.60% | 22 |
|  | $800.00 | 3.80% | 6.20% | 16 |
|  | $1,000.00 | 2.30% | 7.70% | 13 |
|  | $1,200.00 | 0.80% | 9.20% | 11 |
|  | **$1,300.00** | **0.00%** | 10.00% | 10 |
|  | $1,400.00 | — | 10.80% | 9 |
|  | $1,500.00 | — | 11.50% | 9 |
|  | $1,800.00 | — | 13.80% | 7 |
|  | $2,000.00 | — | 15.40% | 6 |
|  | $2,500.00 | — | 19.20% | 5 |
| *****Break-even price of beads  **Rows where baseline infection risk is lower than the ARR are shown as not clinically feasible (—) because infection risk cannot be negative | | | | |
